# Transfusion of CXCR4-Primed Endothelial Progenitor Cells Reduces Cerebral Ischemic Damage and Promotes Repair in db/db Diabetic Mice

**DOI:** 10.1371/journal.pone.0050105

**Published:** 2012-11-21

**Authors:** Ji Chen, Jianying Chen, Shuzhen Chen, Cheng Zhang, Liangqing Zhang, Xiang Xiao, Avik Das, Yuhui Zhao, Bin Yuan, Mariana Morris, Bin Zhao, Yanfang Chen

**Affiliations:** 1 Department of Pharmacology & Toxicology, Boonshoft School of Medicine, Wright State University, Dayton, Ohio, United States of America; 2 Clinical Research Center and Department of Neurology, the Affiliated Hospital of Guangdong Medical College, Zhanjiang, Guangdong, People’s Republic of China; 3 Department of Neurology, the First Affiliated Hospital of Guangxi Medical University, Nanning, Guangxi, People’s Republic of China; University of Florida, United States of America

## Abstract

This study investigated the role of stromal cell-derived factor-1α (SDF-1α)/CXC chemokine receptor 4 (CXCR4) axis in brain and endothelial progenitor cells (EPCs), and explored the efficacy of CXCR4 primed EPCs in treating ischemic stroke in diabetes. The db/db diabetic and db/+ mice were used in this study. Levels of plasma SDF-1α and circulating CD34+CXCR4+ cells were measured. Brain SDF-1α and CXCR4 expression were quantified at basal and after middle cerebral artery occlusion (MCAO). In *in vitro* study, EPCs were transfected with adenovirus carrying null (Ad-null) or CXCR4 (Ad-CXCR4) followed with high glucose (HG) treatment for 4 days. For pathway block experiments, cells were pre-incubated with PI3K inhibitor or nitric oxide synthase (NOS) inhibitor for two hours. The CXCR4 expression, function and apoptosis of EPCs were determined. The p-Akt/Akt and p-eNOS/eNOS expression in EPCs were also measured. In *in vivo* study, EPCs transfected with Ad-null or Ad-CXCR4 were infused into mice via tail vein. On day 2 and 7, the cerebral blood flow, neurologic deficit score, infarct volume, cerebral microvascular density, angiogenesis and neurogenesis were determined. We found: 1) The levels of plasma SDF-1α and circulating CD34+CXCR4+ cells were decreased in db/db mice; 2) The basal level of SDF-1α and MCAO-induced up-regulation of SDF-1α/CXCR4 axis were reduced in the brain of db/db mice; 3) Ad-CXCR4 transfection increased CXCR4 expression in EPCs and enhanced EPC colonic forming capacity; 4) Ad-CXCR4 transfection prevented EPCs from HG-induced dysfunction (migration and tube formation) and apoptosis via activation of PI3K/Akt/eNOS signal pathway; 4) Ad-CXCR4 transfection enhanced the efficacy of EPC infusion in attenuating infarct volume and promoting angiogenesis and neurogenesis. Our data suggest that Ad-CXCR4 primed EPCs have better therapeutic effects for ischemia stroke in diabetes than unmodified EPCs do.

## Introduction

Diabetes is a risk factor for stroke, which are the nation’s second leading cause of death and the leading cause of long-term disability. In diabetic patients, ischemic cerebral damage is exacerbated and the outcome is poor. The responsible mechanisms might include microvascular rarefaction, reduced collateralization and impaired angiogenesis. Endothelial progenitor cells (EPCs) are believed to play an important role in maintaining endothelial integrity and vascular homeostasis and to participate in angiogenesis which represents an important endogenous tissue repair mechanism [1], [2]. Accumulating evidence show that circulating EPCs are reduced in number and impaired in function in diabetic patients and animals [3]–[5]. Studies on ischemic brain, heart and limbs indicate that transfusion of EPCs is able to reduce tissue injury, promotes angiogenic repair and functional recovery [3], [6], [7]. These positive results provide a good rationale for using EPCs to treat ischemic stroke in diabetes.

The stromal cell-derived factor-1α (SDF-1α)/CXC chemokine receptor 4 (CXCR4) axis is believed to play an important role in recruiting progenitor cells into ischemic tissue [8]–[10] and triggers many intracellular proliferation and anti-apoptosis signals, such as mitogen-activated protein kinase (MAPKs), phosphatidylinositol-3-kinase (PI3K) and the serine/threonine kinase Akt [11]. Therefore, it is a potential target for promoting repair in wound and ischemic injury. Recent studies on ischemic heart and limbs have shown that a combination of SDF-1α/CXCR4 over-expression and stem cell transfusion represents an attractive regime for treating ischemic diseases. SDF-1α pretreatment increases the therapeutic potential of EPC transfusion in a mouse model of hindlimb ischemia [12]. Over-expression of CXCR4 in mesenchymal stem cells enhances *in vivo* engraftment into the ischemic heart and subsequently improves functional recovery via augmenting myoangiogenesis [13]. When compared to low-CXCR4-expressing EPCs, administration of high-CXCR4-expressing EPCs further increases capillary density and promotes blood flow recovery in ischemic hindlimbs [14]. However, there is little information on EPCs-based therapy for ischemic stroke in diabetes.

In this study, we investigated whether the SDF-1α/CXCR4 signal pathway is dysregulated in the brain of db/db diabetic mice. In EPC cultures, we determined the role of CXCR4/PI3K/Akt/eNOS signaling pathway and high glucose (HG) in EPC function and survival. Furthermore, we tested the hypothesis that transfusion of Ad-CXCR4 primed EPCs is more effective on treating ischemic stroke in db/db mice.

## Material and Methods

### Animal Experimental Design

**Figure 1:**
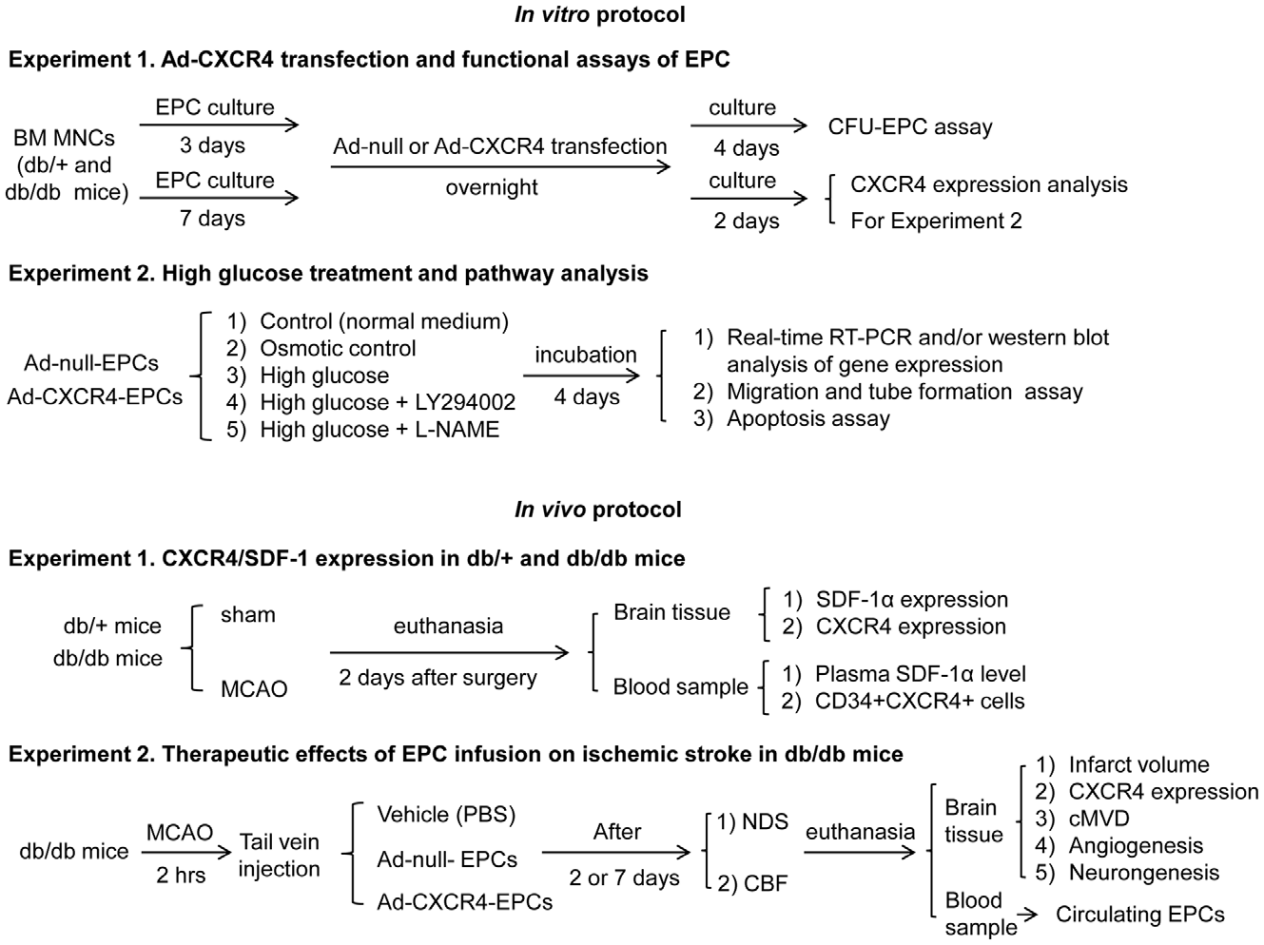
Experimental protocols. The flow diagrams briefly describe the *in vitro* and *in vivo* protocols.

Adult male db/db diabetic mice (C57BL6/J) and their age matched (8–10 weeks) controls (db/+) were used for the study (Jackson Laboratories, Bar Harbor, Maine). The general characteristics of db/+ and db/db mice are summarized in Table 1. The db/db mice possess an inactivating mutation of the gene-encoding leptin receptor and subsequently develop obesity, hyperglycemia and insulin resistance resembling adult-onset diabetes mellitus. Therefore, the db/db mice are commonly used mouse model for type 2 diabetes [15]. The level of fasting plasma glucose was measured after 16 hours fasting by an Accu-Check Advantage Blood Glucose Monitor (Roche Diagnostic, Indianapolis, IN). All experimental protocols (Figure 1) were approved by the Laboratory Animal Care and Use Committees at both Wright State University and Guangdong Medical College in accordance to the Guide for the Care and Use of Laboratory Animals issued by the National Institutes of Health.

**Table 1:** General Characteristics of db/+ and db/db Mice.

Variants	db/+ (n = 34)	db/db (n = 90)
Age (W)	8.2±0.5	8.5±0.4
B.W. (g)	27.9±0.6	44.8±1.2**
Blood glucose (mg/dl)	123.6±12.5	421.5±10.4**

Data are means ± SE.

* *P*<0.05, **P<0.01, compared with db/+. B.W.: Body weight.

#### Protocol one

For exploring whether SDF-1α/CXCR4 axis is dysregulated in the brain of diabetes (at basal and after ischemic stroke), db/db (n = 12) and db/+ mice (n = 12) were randomly assigned to middle cerebral artery occlusion (MCAO) or sham surgery group. MCAO surgery was performed to induce focal cerebral ischemic stroke as we previously described [3], [16]. Mice were euthanized 48 hours after surgery, and the brain tissues were immediately removed. The ischemic ipsilateral and contralateral side hemispheres were dissected for analysis of SDF-1α and CXCR4 expression. For real-time RT-PCR analysis, tissues were immediately harvested into 1 ml tubes containing 0.5 ml RNAlater (Qiagen, CA) and cut into small pieces (<0.5 cm^3^). After overnight, tissues were transferred to store at −80°C. For western blot analysis, tissues were immediately harvested into tubes and put on dry ice before they were transferred to a −80°C freezer. Blood samples were collected for analysis the level of plasma SDF-1α and circulating CD34+CXCR4+ cells.

#### Protocol two

For determining the therapeutic efficacy of Ad-CXCR4 primed EPCs on ischemic stroke in diabetes, bone marrow (BM) (donated from db/+ mice, n = 16) derived EPCs were cultured for 7 days and then transfected with adenovirus (Ad) carrying null (Ad-null-EPCs) or CXCR4 gene (Ad-CXCR4-EPCs) overnight. After transfection, EPCs were continuously cultured for another 2 days to expansion. After that, the cells were harvested, counted for injection. The db/db mice (n = 72) were subjected to MCAO surgery (under anesthesia by inhaling 2.5% isoflurane) and randomly assigned to different treatment groups: vehicle (phosphate buffered solutions, PBS), Ad-null-EPCs and Ad-CXCR4-EPCs. Pain and discomfort were minimized by an initial injection of Buprenorphine (0.1 mg/kg, s.c) followed with another two injections every 12 hours. Mice were injected via the tail vein with EPCs (2×10^5^ cells/100 µl in PBS) or the same volume of PBS two hours after MCAO [3], [17]. To label the new generated cells, mice were injected with bromodeoxyuridine (BrdU, 65 µg/g/day, i.p.) immediately after MCAO surgery until the day of experimental endpoint [18]. Neurologic motor function was determined and cerebral blood flow (CBF) was measured (under anesthesia by inhaling 2.5% isoflurane) right before mice (n = 12/group/time point) were euthanized on day 2 or 7. Blood samples were taken from the heart for analyzing the level of circulating EPCs under deep anesthesia (pentobarbital, 150 mg/kg body weight) [3]. For real-time RT-PCR analysis of CXCR4 expression (n = 6/group/time point), brain tissues of the ischemic hemisphere were immediately harvested as described in Protocol one. For histological analysis (n = 6/group/time point), mice were perfused with PBS and 4% paraformaldehyde (PFA). Then, brain tissues were fixed in 4% PFA plus 30% sucrose for 3 days. Fixed brains were then cut into sequential coronary sections (20 µm) and divided into four wells for Fluoro-Jade staining analysis of infarct volume, and immunohistological analysis of cerebral microvascular density (cMVD), angiogenesis and neurongenesis.

### Enzyme-linked Immunosorbent Assay (ELISA) for SDF-1α

The plasma level of SDF-1α was measured by ELISA methods [19]. Briefly, mouse plasma was collected and detected by mouse CXCL12/SDF-1α ELISA kit (R&D systems, MN). Absorbance was read at 450 nm.

### Real-time Reverse Transcription Polymerase Chain Reaction (RT-PCR)

The levels of SDF-1α and CXCR4 of the brain tissues were determined using real-time RT-PCR methods [20]. Brain total mRNAs were isolated using RNeasy Mini kit (Qiagen, CA) and reverse-transcripted with the high capacity cDNA archive kit (Qiagen). The real-time PCR was run using SYBR Green reagents (Qiagen). The primer sequences: CXCR4 (5′-TTT CAG CCA GCA GTT TCC TT-3′ and 5′-TCA GTG GCT GAC CTC CTC TT-3′); SDF-1α (5′-CCC GGA TCC ATG AAC GCC AAG GTC GTG-3′ and 5′-AGA GCT GGG CTC CTA CTG TGC GGC CGC GGG-3′). β-actin was chosen for housekeeping gene for normalizing the data of gene expression.

### Bone Marrow EPC Culture and Characterization

EPCs were generated from BM mononuclear cells (MNCs) as we previously reported [3], [21]. In brief, BM was flushed out from tibias and femurs and BM MNCs were isolated by using density gradient centrifuge method. BM MNCs isolated from db/+ and db/db mice were counted and plated (1×10^6^ cells/well) on fibronectin-coated 24-well plates (BD Bioscience, San Jose, CA, USA) and then grown in endothelial cell basal medium-2 (EBM-2) supplemented with 5% FCS containing EPC growth cytokine cocktail (Lonza, Walkersville, MD, USA). After 3 days in culture, non-adherent cells were removed by washing with PBS. Thereafter, culture medium was changed every 2 days. EPCs were characterized by double staining with Di-LDL and BS-Lectin, and flow cytometric analysis of specific EPC surface markers (CD34 and VEGFR2) on day 7.

### Ad-CXCR4 Preparation and Transfection

The Ad-CXCR4 was kindly provided by Dr. Yigang Wang in the Department of Pathology and Experimental Medicine at University of Cincinnati. The rat CXCR4 cDNA (MGC-36266) was purchased from ATCC (American Type Culture Collection) and sub-cloned in the BglII and HindIII sites of plasmid pEGFP-C1 (Clonetech) by polymerase chain reaction (PCR) technology. The identity of the gene confirmed by sequencing was subsequently cloned into the same restriction sites on the shuttle vector pAdTrack-CMV which contains the enhanced green fluorescence protein (EGFP) expression cassette. Recombinant adenovirus expresses CXCR4 and green fluorescent protein (GFP) under cytomegalovirus (CMV) promoter. EPCs were transfected with Ad-null or Ad-CXCR4 as previously describes [13]. Briefly, EPCs cultured in six-well plates with 75% confluence were incubated with 1×10^7^ infectious units of Ad-null or Ad-CXCR4 in non-FCS medium overnight. The viruses were removed and the medium was replaced with fresh medium with FCS in the following day. Cells were continuously cultured for 2 days to reach confluence for harvest. CXCR4 expression in EPCs was confirmed by real-time RT-PCR and western blot. The percentage of CXCR4+ EPCs was analyzed by a flow cytometer (Accuri C6 flow cytometer, Inc. Ann Arbor, MI) after staining EPCs with anti-CXCR4 (CXCR4-PE, eBioscience, San Diego, CA). CXCR4+ EPCs (%)** = **events of CXCR4+ EPCs/total events of EPCs×100%.

### Colony Forming Unit Counts of EPCs

EPCs from db/+ and db/db mice were cultured in EBM-2 medium and seeded in six-well plates precoated with fibronectin. After 3 days in culture, non-adherent cells were removed by washing with PBS and the adherent cells were transfected with Ad-null or Ad-CXCR4 (1×10^7^ infectious units) overnight. On day 7 (4 days after transfection), the numbers of colony formation unit (CFU) were counted by visual inspection with an inverted microscope (Nikon TMS, Japan). A colony of EPCs was defined as a central core of round cells with elongated sprouting cells at the periphery, as previously reported [22].

### High Glucose Experiments on EPCs

The HG (25 mmol/L) medium which corresponds to 350–450 mg/dl of plasma glucose levels in diabetic patients was used in the present study [23]. Mannitol (19.5 mM) was used for osmotic control as previous reports [24]–[25]. Ad-null-EPCs or Ad-CXCR4-EPCs were cultured in HG, mannitol or basal EPC medium supplemented with SDF-1α (100 ng/ml) for 4 days before functional assays. The medium were changed every two days to maintain HG level [26] and the glucose level of the culture supernatant was daily monitored by an oxidase-based colorimetric method [27] during the HG experiments. For blocking experiments, cells were pre-incubated with PI3K inhibitor (LY294002, 20 µM, Cell Signaling) or NOS inhibitor (*N* ^G^-nitro-arginine methyl ester, L-NAME, 1 mM, Cell Signaling Technology, Inc. MA) for two hours [28].

### EPC Migration and Tube Formation Assays

EPC migration and tube formation were evaluated by using Boyden chamber (Chemicon, Rosemont, IL) and tube formation assay kit (Chemicon) methods as we previously described [3]. For migration, EPCs (2×10^4^ cells) were placed into upper compartment of the Boyden chamber (Chemi-con, Rosemont, IL, USA) with 50 ng/ml vascular endothelial growth factor (VEGF) and 100 ng/ml stromal cell-derived factor-1 (SDF-1) in the lower compartment. After 24 hours, the EPCs which migrated across the membrane were counted under an inverted light microscope, quantified and averaged by examining ten random microscopic fields (magnification,×200). For tube formation, ECMatrix’ solution was thawed on ice overnight, mixed with 10×ECMatrix’ diluents and placed in a 96-well tissue culture plate at 37°C for one hour to allow the matrix solution to solidify. EPCs were re-plated (1×10^4^ cells/well) on top of the solidified matrix solution and incubated for 24 hours at 37°C. Tube formation was evaluated with an inverted light microscope and defined as a tube structure exhibiting a length 4 times its width [29]. Five independent fields were assessed for each well, and the average number of tubes per field (magnification, ×200) was determined. During migration and tube formation assays, EPCs were cultured in the basal EPC medium as previously described [30].

### EPC Apoptosis Assay

After 4 days’ culture in HG medium, EPCs were harvested for apoptosis analysis by using Alexa Fluor 488 annexin V/dead cell apoptosis kit (Molecular Probes, invitrogen, Carlsbad, CA). Briefly, cells were resuspended in annexin-binding buffer, and then incubated with annexin V and propidium iodide (PI) for 15 min at room temperature (RT). The apoptotic EPCs were recognized as PI−/Annexin V+ cells. The percentage of apoptosis was analyzed by flow cytometer.

### Western Blot Analysis

Gene expression of SDF-1α, CXCR4, eNOS, Akt, p-eNOS or p-Akt of the brain tissue or EPCs was determined [28], [31]. Proteins were isolated with lysis buffer (Roche Diagnostic) containing protease inhibitor. The proteins were subjected to SDS-PAGE electrophoresis and transferred onto nitrocellulose membranes. The membranes were blocked by incubating with 5% dry milk and Tris-buffered saline for one hour, and then incubated with antibodies against SDF-1α (1∶200; R&D systems), CXCR4 (1∶100, AnaSpec Inc. CA), Akt (1∶1000, Cell Signaling Technology), eNOS (1∶1000, Cell Signaling Technology), p-Akt, (1∶1000, Cell Signaling Technology), or p-eNOS (1∶1000) at 4°C overnight. β-actin (1∶4000, Sigma, MO) was used to normalize protein loading. After being washed thoroughly, membranes were incubated with horseradish peroxidase (HRP) conjugated IgG (1∶40000, Jackson Lab) for one hour at RT. Blots were then developed with enhanced chemiluminescence developing solutions and quantified.

### Flow Cytometry Analysis of Circulating EPCs and CD34+CXCR4+ Cells

The level of circulating EPCs was determined by flow cytometry as a previous study [3]. Briefly, circulating MNCs were isolated by density gradient centrifugation and stained with anti-mouse CD34-PE (AbD Serotec, Raleigh, NC) and VEGFR2-PE-Cy7 (BD, Bioscience) antibodies for 30 min at RT. For detecting CD34+CXCR4+ cells, circulating MNCs were stained with CD34-FITC (AbD Serotec) and CXCR4-PE (eBioscience, San Diego, CA) antibodies. The levels of circulating EPCs and CD34+CXCR4+ cells were expressed as cells/ml blood. Isotype (IgG) antibodies were used as respective negative controls for data calibration.

### Functional Evaluation of Neurological Deficits

The neurological deficit scores were evaluated on day 2 or 7 after EPC treatment for functional determination of therapeutic efficacy in each group. The 5-point scale method was previously described [16], [32]. The five points are: 0, normal motor function; 1, flexion of contralateral torso and forelimb upon lifting the whole animal by the tail; 2, circling to the contralateral side but normal posture at rest; 3, leaning to the contralateral side at rest; 4, no spontaneous motor activity. The neurologic behavior of mice was scored by an investigator who was unaware of animal grouping.

### Measurement of Cerebral Blood Flow

On day 2 or 7 following EPC transfusion, the relative CBF in the peri-infarct area was determined as described previously [33], [34] with slight modification. Briefly, mouse was anesthetized with 2.5% isoflurane and placed on a stereotaxic apparatus. An incision was made in the scalp to expose the skull. The volumes of CBF at the peri-infarct site of ischemic ipsilateral area (2 mm posterior, 6 mm lateral to bregma) and contralateral site (2 mm posterior, 6 mm contralateral to bregma, serves as basal normal level for calibration) were sequentially determined using a laser Doppler flowmeter (PF2B, Perimed, Sweden) with a fiberoptic probe (tip diameter 0.5 mm). To minimize variability, the CBF was recorded at each site for at least 5 minutes. The averaged volume over 5 minutes was used to represent CBF for each site. The relative CBF was calculated using the formula: relative CBF = CBF of ipsilateral side/CBF of contra-lateral side x100%. The person who performed CBF measurements was unaware of the information of animal grouping.

### Measurement of Infarct Volume and Cerebral Microvascular Density

As we previously described [3], [16], cerebral ischemic damage and the cMVD in peri-infarct area were revealed by staining brain coronal sections (20 µm) with Fluoro-Jade (0.001%, Histo-chem, Jefferson, AR, USA) and CD31 (1∶50, Invitrogen), respectively. Infarct volume and cMVD were quantified using the Image J software (NIH).

### Analysis of Angiogenesis and Neurongenesis

Angiogenesis and neurongenesis in peri-infarct area were determined by using double immunofluorescence staining with BrdU and either cell-specific biomarker CD 31 (endothelial cells, ECs), neuronal nuclei (NeuN), or glial fibrillary acidic protein (GFAP) [18]. Specifically, brain coronal sections were incubated with BrdU antibody (1∶50, Abcam, MA, USA), followed by incubation with cell-specific antibodies: CD31 (1∶50, BD Biosciences), GFAP (1∶400, Chemicon), or NeuN (1∶200, Chemicon) overnight at 4°C. Next, brain sections were reacted with FITC (for BrdU) or Cy3 (for cell specific markers) conjugated secondary antibodies (1∶250, Invitrogen) for 30 min at RT in the dark. The labeled ECs (BrdU+CD31+), neurons (BrdU+NeuN+) and glial cells (BrdU+GFAP+) in the peri-infarct area of each section were counted under 6 random fields (200×). The average of five sections from rostral to caudal represented the data for each brain. The newly generated cells were counted by an investigator who was unaware of animal grouping.

### Statistical Analysis

All data, excepting neurologic deficit scores, are presented as mean ± SE. The neurologic deficit scores were expressed as median (range). The neurological deficit scores among different groups were compared by the Kruskal–Wallis test. When the Kruskal–Wallis test showed a significant difference, the Mann–Whitney U-tests were applied. For the rest measurements, comparisons for two groups were performed by the student’s t test. Multiple comparisons were analyzed by one- or two-way ANOVA. For all tests, a *P*-value <0.05 was considered significant.

## Results

### Baseline Characterization of Animals

The characterizations of blood glucose, age and body weight in db/db and db/+ mice used in this study are presented in Table 1. As expected, the type 2 diabetic db/db mice had higher plasma glucose and body weight as compared with age-matched db/+ control mice. In protocol two, db/db mice were subjected to MCAO surgery (blood flow <75% of baseline) and randomized to vehicle, Ad-null-EPC or Ad-CXCR4-EPC infusion groups. There was no difference in body weight and blood glucose among different treatment groups (Table 2).

**Table 2:** Baseline Characteristics of db/db Mice in Different Groups.

Groups	B.W. (g)	Blood glucose (mg/dl)
Vehicle, 2 day	48.2±2.1	428.5±10.4
Vehicle, 7 day	47.9±2.2	425.8±11.2
Ad-null-EPCs, 2 day	43.6±1.5	421.6±12.4
Ad-null-EPCs, 7 day	42.2±1.5	418.4±11.8
Ad-CXCR4-EPCs, 2 day	46.9±1.6	424.8±12.2
Ad-CXCR4-EPCs, 7 day	46.6±1.1	421.5±12.4

Data are means ± SE. n = 6/group. B.W.: Body weight.

### The Levels of Plasma SDF-1α and Circulating CD34+CXCR4+ Cells are Reduced in db/db Mice

The level of plasma SDF-1α was significantly lower in db/db mice (1.3±0.14 and 1.8±0.15 pg/ml, *P*<0.05, db/db *vs.* db/+ mice, n = 9/group). The level of circulating CD34+CXCR4+ cells was reduced in db/db mice (260±14 and 712±42 cells/ml, *P*<0.01, db/db *vs.* db/+ mice, n = 9/group).

### The Expression of SDF-1α/CXCR4 Axis is Dysregulated in the Brain of db/db Mice at Basal and Following Ischemic Stroke

**Figure 2:**
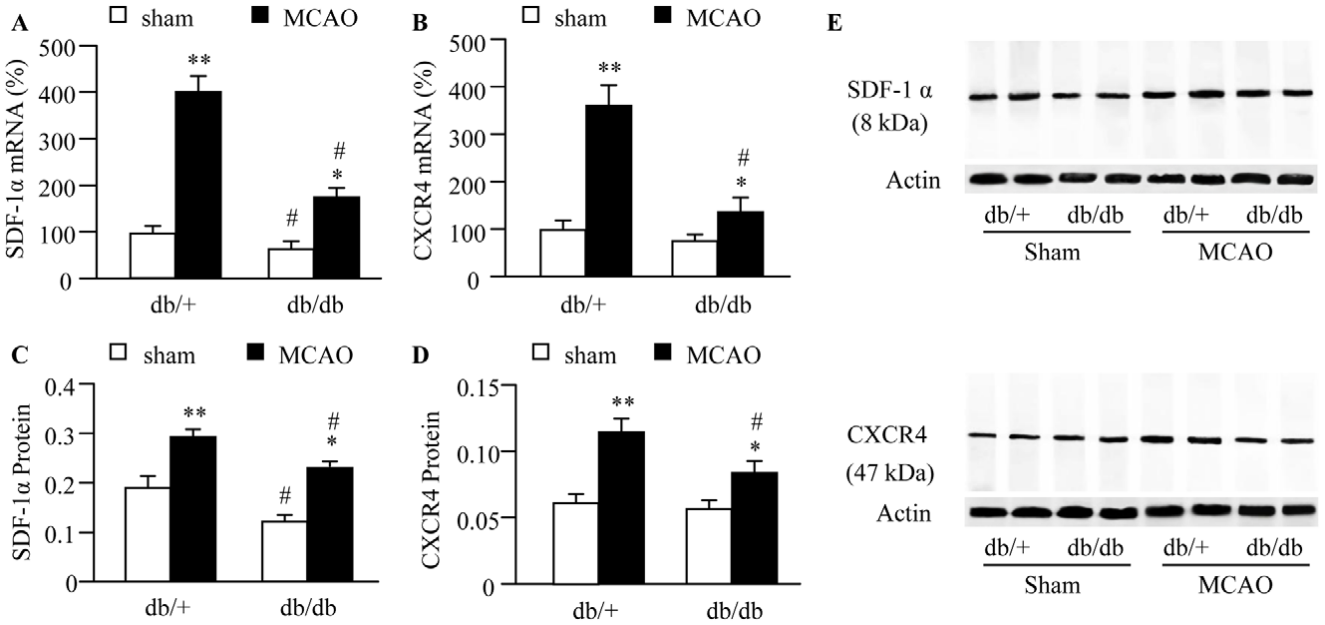
SDF-1α/CXCR4 expression in the brain of db/db mice at basal and in response to ischemia. (A) SDF-1α mRNA expression. (B) CXCR4 mRNA expression. (C) SDF-1α protein expression. (D) CXCR4 protein expression. (E) Representative protein bands of SDF-1α and CXCR4. *P<0.05, **P<0.01 *vs.* sham; ^#^ *P*<0.05 *vs.* db/+, n = 5/group in mRNA analysis, n = 6/group in protein analysis.

At basal, the db/db diabetic mice had less expression of SDF-1α in the brain tissue at both mRNA and protein levels (*P*<0.05; Figure 2A and C) with no significant difference in CXCR4 expression (*P*>0.05; Figure 2B and D). The levels of brain SDF-1α and CXCR4 in the ischemic ipsilateral hemisphere of brain tissue were up-regulated in both db/db and db/+ mice 48 hours following MCAO (*P*<0.05 or 0.01). However, the up-regulations of SDF-1α and CXCR4 were less in db/db mice (*P*<0.05; Figure 2). The levels of brain SDF-1α and CXCR4 in the contralateral hemisphere was unaffected (data not shown).

### EPC Characterization and CXCR4 Expression in EPCs

**Figure 3:**
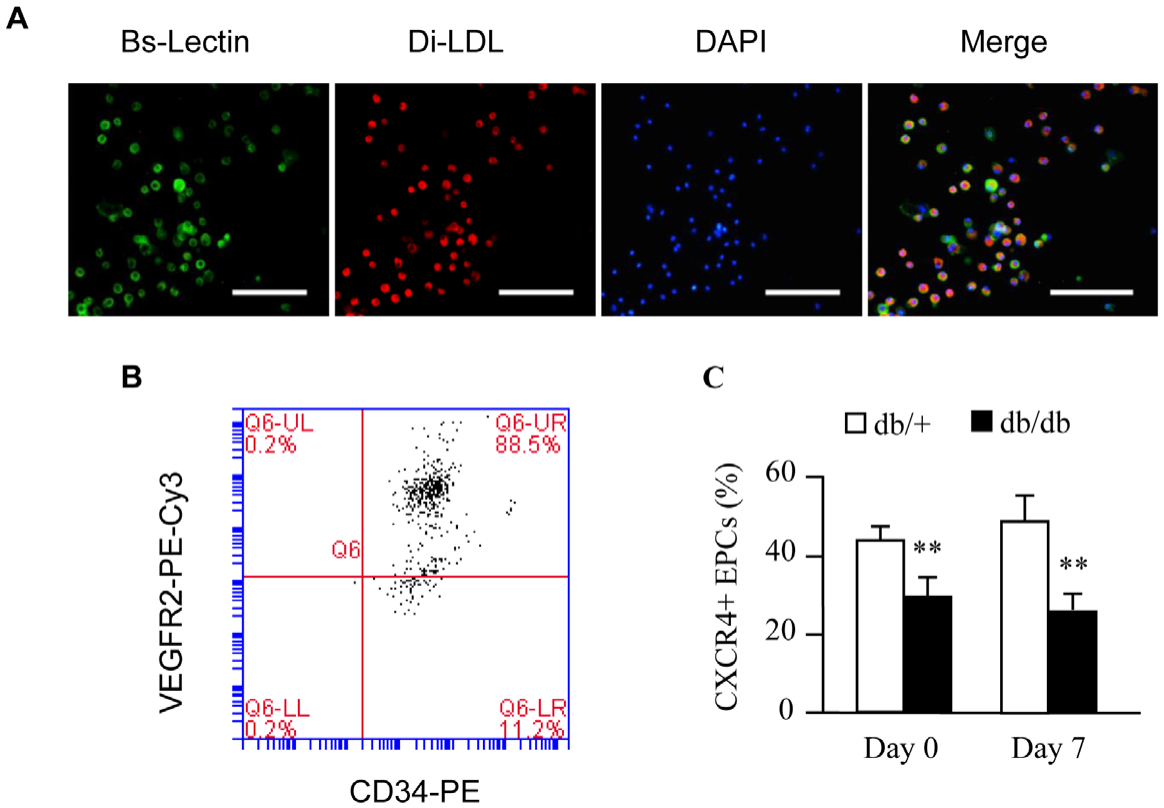
Characterization of bone marrow derived EPCs. (A) Representative pictures showing cultured EPCs by double staining analysis. Red: Di-LDL up-taking; Green: Bs-Lectin staining; Blue: DAPI (nuclear); Yellow: Di-LDL and Bs-Lectin positive cells defined as EPCs. Scale bar: 75 µm. (B) Representative flow plot showing the percentage of CD34/VEGFR2 expression in EPCs. At the end of EPC culture, cells were stained with CD34 and VEGFR2, and analyzed by flow cytometry. EPCs were defined as CD34+VEGFR+ cells. (C) Summarized data of CXCR4 expressing EPCs after day 0 and 7 days’ culture. **P<0.01 *vs.* db/+, n = 6/group.

BM derived EPCs were defined as cells up-taking Di-LDL and binding with Bs-Lectin, as well as cells expressing CD34/VEGFR2 using flow cytometric method (Figure 3A and B). At the end of EPC culture (7 days), the percentage of CD34+VEGFR2+ EPCs was 89±3.5% (n = 6, Figure 3B). In addition, 7 days’ culture of EPCs did not cause any change of the percentage of CXCR4+ EPCs (Day 0 *vs.* Day 7; *P*>0.05). The CXCR4+ EPCs was lower in db/db mice (*P*<0.01; Figure 3C).

### Ad-CXCR4 Transfection Increases CXCR4 Expression and Colony Forming Capacity of EPCs

**Figure 4:**
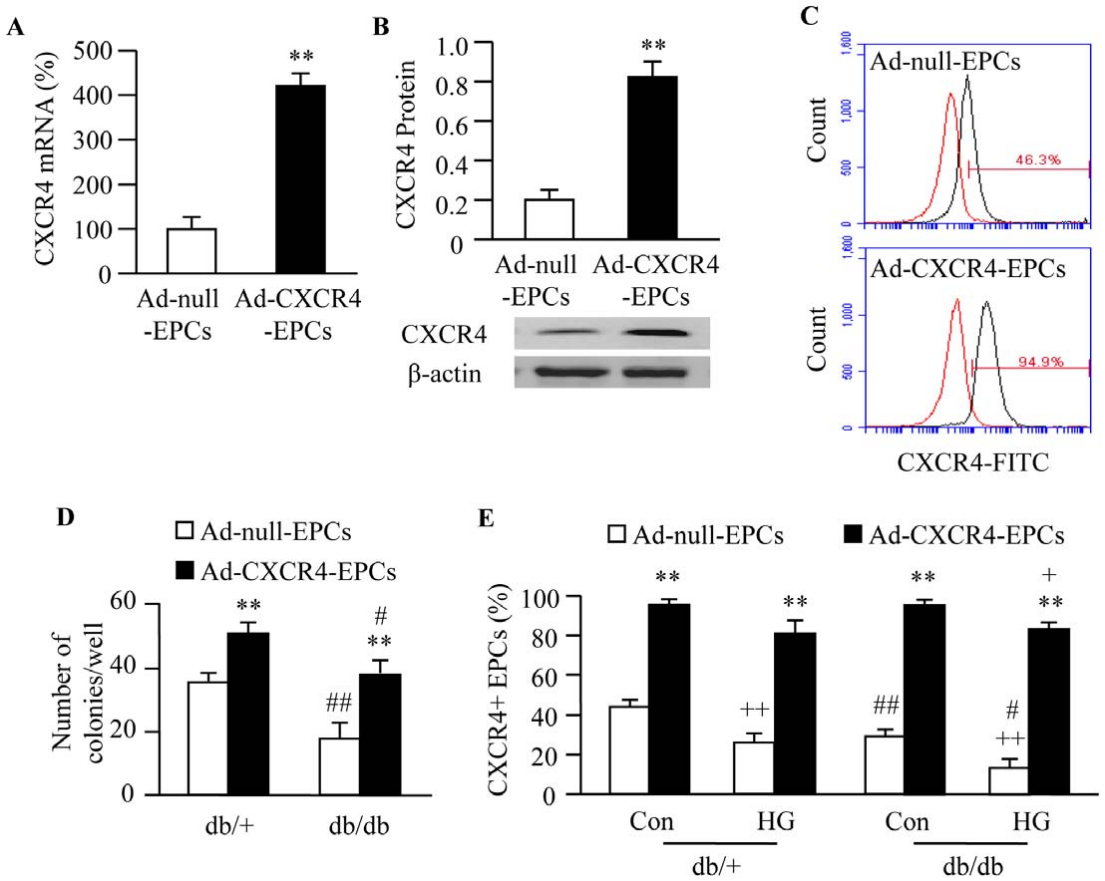
CXCR4 expression and colony forming capacity of EPCs. CXCR4 mRNA expression (A) and protein expression (B) in EPCs after Ad-CXCR4 transfection. (C) Representative histogram distributions of flow data showing the percentage of CXCR4+ EPCs in Ad-null-EPCs and Ad-CXCR4-EPCs. The red line is the IgG isotype control. (D) Summarized data on the colony forming units of EPCs. (E) Summarized data showing the expression of CXCR4 in EPCs from both db/+ and db/db mice with or without HG (25 mM) treatment. **P<0.01 *vs.* Ad-null-EPCs; ^+^ *P*<0.05, ^++^ *P*<0.01 *vs.* Con;^ #^ *P*<0.05,^ ##^ *P*<0.01 *vs.* db/+, n = 6/group. Con: control (basal medium); HG: high glucose medium; Ad-null-EPCs: EPCs transfected with Ad-null; Ad-CXCR4-EPCs: EPCs transfected with Ad-CXCR4.

Real-time PCR and western blot analyses showed that Ad-CXCR4 transfection up-regulated CXCR4 expression in EPCs by about two-fold at both mRNA and protein levels (*P*<0.01; Figure 4A and B). Flow cytometric result showed that Ad-CXCR4 transfection significantly increased the percentage of CXCR4+ EPCs (*P*<0.01; Figure 4C). The number of CFUs was decreased in EPCs from db/db mice (*P*<0.05 or 0.01; Figure 4D). Ad-CXCR4 transfection increased CFUs of EPCs from both db/+ and db/db mice (*P*<0.01; Figure 4D).

### Ad-CXCR4 Transfection Protects EPCs from HG-induced Dysfunction and Apoptosis by Activating its Downstream PI3K/Akt/eNOS Signaling Pathway

**Figure 5:**
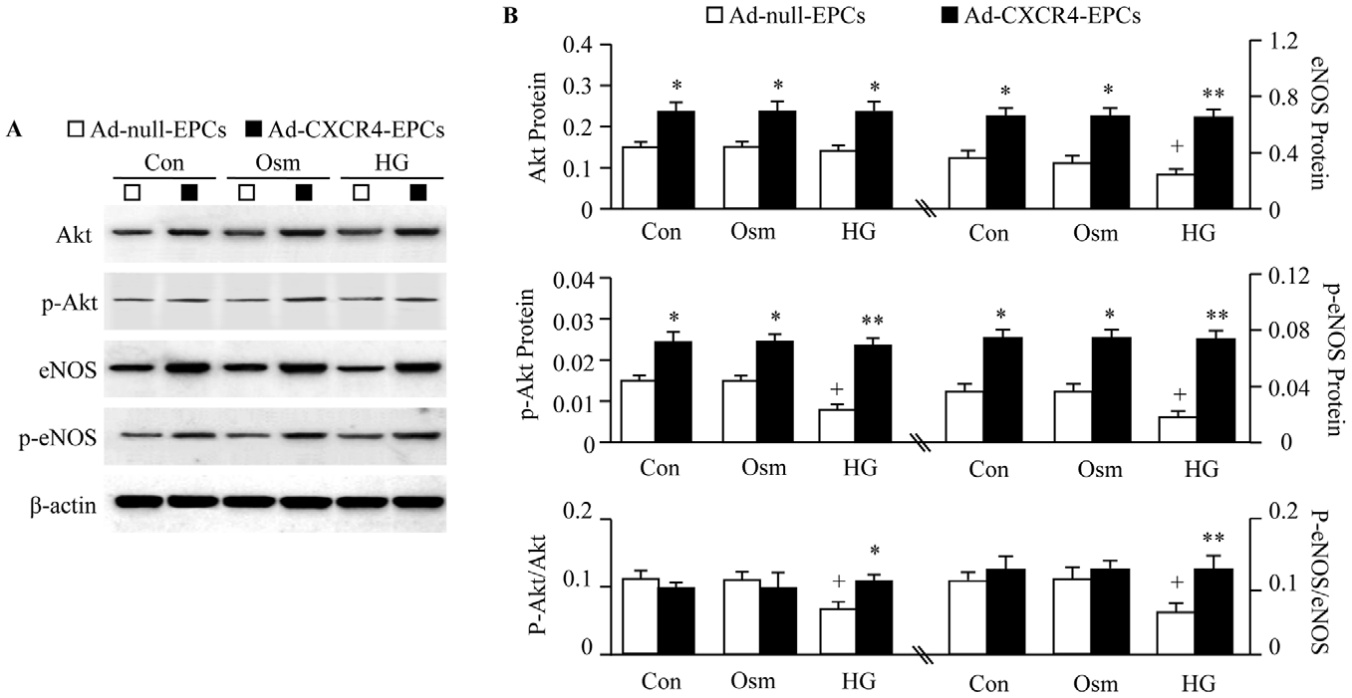
Ad-CXCR4 transfection protects down-regulation of Akt/eNOS activation in EPCs induced by HG. (A) Representative western blot bands showing Akt/eNOS and p-Akt/p-eNOS expression in different treatment groups. The molecular weights are 60 kDa for Akt and p-Akt, and 140 kDa for eNOS and p-eNOS. (B) Summarized data on Akt/eNOS and p-Akt/p-eNOS expression in EPCs of different treatment groups, *P<0.05, **P<0.01 *vs.* Ad-null-EPCs; ^++^ *P*<0.01 *vs.* Con or Osm. Con: control (basal medium); Osm: osmotic control; HG: high glucose medium; p-Akt: phosphorylated Akt; p-eNOS: phosphorylated eNOS; PI3K: phosphatidylinositol-3-kinase; NOS: nitric oxide synthase; Ad-null-EPCs: EPCs transfected with Ad-null; Ad-CXCR4-EPCs: EPCs transfected with Ad-CXCR4.

**Figure 6:**
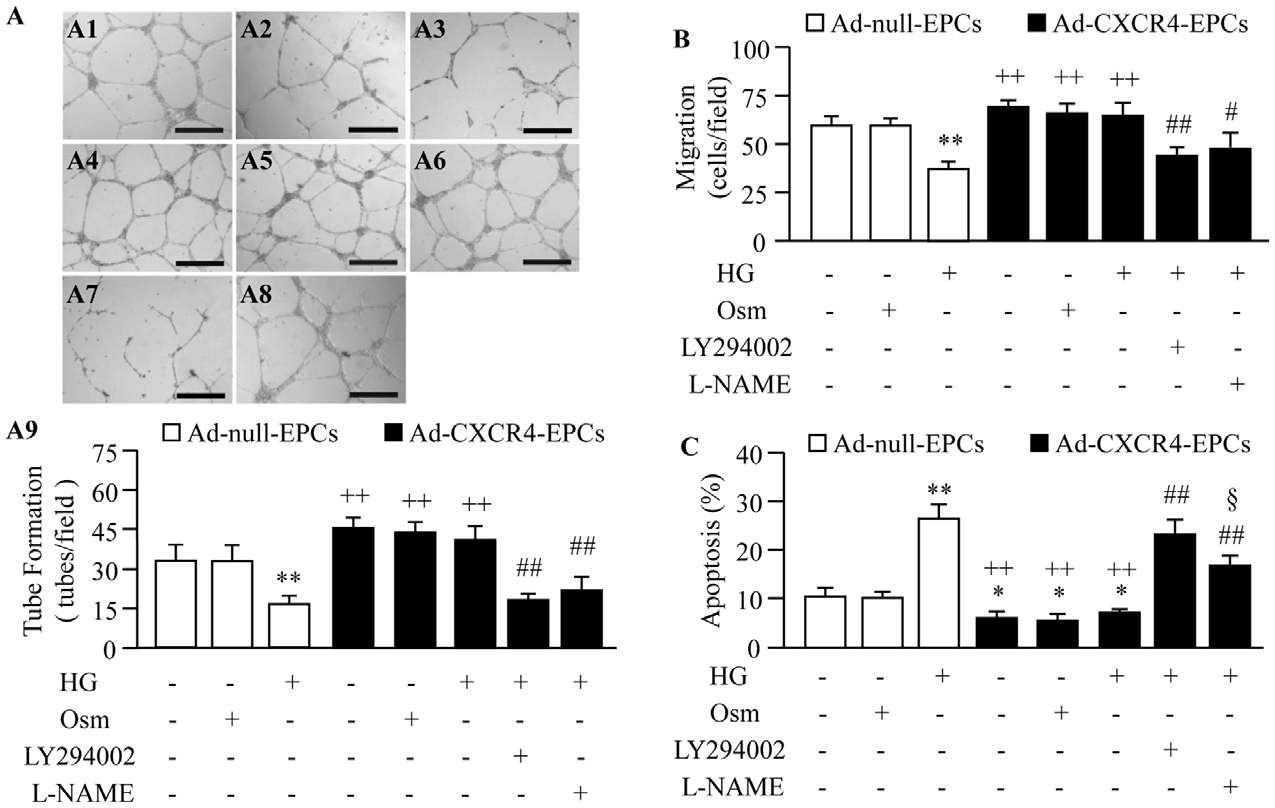
Ad-CXCR4 transfection protects EPCs from HG-induced dysfunction and apoptosis via activating Akt/eNOS pathways. Representative tube formation pictures (A1–A8) and summarized data (A9) in different treatment groups. A1: Ad-null-EPCs+Con; A2: Ad-null-EPCs+Osm; A3: Ad-null-EPCs+HG; A4: Ad-CXCR4-EPCs+Con; A5: Ad-CXCR4-EPCs+Osm; A6: Ad-CXCR4-EPCs+HG; A7: Ad-CXCR4-EPCs+HG+LY294002; A8: Ad-CXCR4-EPCs+HG+L-NAME. Scale bar: 600 µm. Summarized data on migration ability (B) and the percentage of EPC apoptosis (C) in different treatment groups. *P<0.05, **P<0.01 *vs.* Ad-null-EPCs or Ad-null-EPCs+Osm; ^++^ *P*<0.01 *vs.* HG+Ad-null-EPCs; ^#^ *P*<0.05, ^##^ *P*<0.01 *vs.* HG+Ad-CXCR4-EPCs; ^§^ *P*<0.05 *vs.* HG+Ad-CXCR4-EPCs+LY294002, n = 6/group. Con: control (basal medium); Osm: osmotic control; HG: high glucose medium; Ad-null-EPCs: EPCs transfected with Ad-null; Ad-CXCR4-EPCs: EPCs transfected with Ad-CXCR4.

HG incubation for 4 days significantly decreased the expression of CXCR4 in EPCs from both db/+ and db/db mice (*P*<0.01; Figure 4E). Ad-CXCR4 transfection increased the expression of CXCR4 in EPCs from both db/+ and db/db mice (*P*<0.01; Figure 4E). HG incubation also impaired EPC function (migration and tube formation, *P*<0.01; Figure 5A–B) and induced EPC apoptosis (*P*<0.01; Figure 5C). Ad-CXCR4 transfection prevented EPCs from HG-induced dysfunction (*P*<0.01; Figure 5A–B) and apoptosis (*P*<0.01; Figure 5C). Meanwhile, the expression of p-Akt/p-eNOS in EPCs was measured. Parallel to the expression of CXCR4, HG induced down-regulation of p-Akt/p-eNOS, whereas did not affect the expression of Akt/eNOS in EPCs. Ad-CXCR4 transfection prevented HG induced down-regulation of p-Akt and p-eNOS and increased the expression of Akt and eNOS in EPCs (Figure 6). Pre-incubation with PI3K inhibitor (LY294002) abolished the effects of Ad-CXCR4 transfection on EPC function and apoptosis (*P*<0.05 or 0.01), Whereas, NOS inhibitor (L-NAME) partially blocked these protective effects (*P*<0.01; Figure 5).

### Infusion of Ad-CXCR4 Primed EPCs Enhances the Efficacy in Increasing the Level of Circulating EPCs and CXCR4 Expression in the Brain

**Figure 7:**
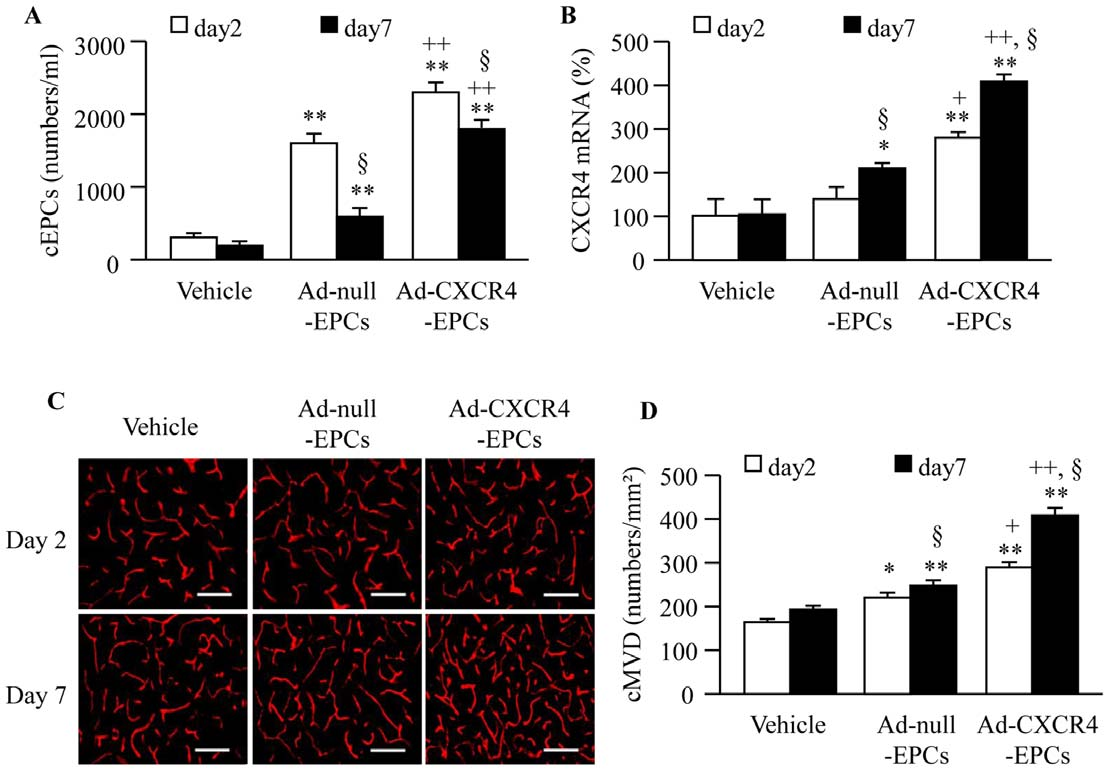
Effects of Ad-CXCR4-EPC infusion on cEPCs, brain CXCR4 expression and cMVD in db/db mice. (A) The level of cEPCs in each therapeutic group. (B) The CXCR4 expression in the brain of db/db mice in each therapeutic group. (C) Representative pictures of cMVD (CD31 immunostaining) in the peri-infarct area. Scale bar: 50 µm. (D) The level of cMVD in the brain sections of db/db mice in each therapeutic group. *P<0.05, **P<0.01 *vs.* vehicle; ^+^ *P*<0.05, ^++^ *P*<0.01 *vs.* Ad-null-EPCs; ^§^ *P*<0.05 *vs.* day 2, n = 6/group. cEPCs: circulating endothelial progenitor cells, cMVD: cerebral microvascular density; Ad-null-EPCs: EPCs transfected with Ad-null; Ad-CXCR4-EPCs: EPCs transfected with Ad-CXCR4.

The db/db mice were treated with EPCs two hours after MCAO surgery. Infusion of Ad-null-EPCs was able to increase the levels of circulating EPCs on day 2 and 7 (*P*<0.01; Figure 7A). Infusion of Ad-CXCR4 primed EPCs further increased the level of circulating EPCs at these time points (*P*<0.01; Figure 7A). Moreover, infusion of Ad-null-EPCs increased CXCR4 expression in the brain of ischemic side on day 7 (*P*<0.05) with no significant change on day 2 (*P*>0.05; Figure 7B). Whereas, infusion of Ad-CXCR4 primed EPCs was more effective to increase CXCR4 expression in the ischemic hemisphere on both day 2 and day 7 (*P*<0.01; Figure 7B).

### Infusion of Ad-CXCR4 Primed EPCs Enhances the Efficacy in Increasing cMVD in the Peri-infarct Area of Ischemic Damage

Infusion of Ad-null-EPCs was able to increase the cMVD in peri-infarct area in db/db mice (Day 2, *P*<0.05; Day 7, *P*<0.01; Figure 7C and D). As expected, transfusion of Ad-CXCR4 primed EPCs could enhance the efficacy (*P*<0.01; Figure 7C and D).

### Infusion of Ad-CXCR4 Primed EPCs Enhances the Efficacy in Increasing Relative CBF, and Decreasing Ischemic Injury and Neurologic Deficit Score

**Figure 8:**
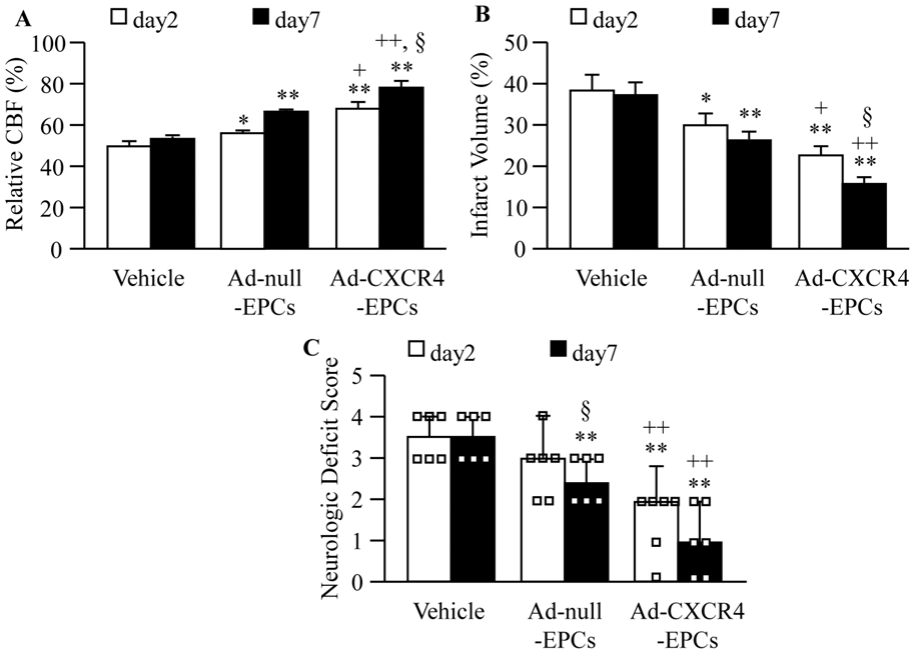
Effects of Ad-CXCR4-EPC infusion on CBF, infarct volume and neurologic deficit score in db/db mice. (A) The relative CBF in peri-infarct area in each therapeutic group. (B) The infarct volume in each therapeutic group. (C) The neurologic deficit scores in each therapeutic group. *P<0.05, **P<0.01 *vs.* vehicle; ^+^ *P*<0.05, ^++^ *P*<0.01 *vs.* Ad-null-EPCs; ^§^ *P*<0.05 *vs.* day 2, n = 6/group. CBF: cerebral blood flow; Ad-null-EPCs: EPCs transfected with Ad-null; Ad-CXCR4-EPCs: EPCs transfected with Ad-CXCR4.

In agreement with the findings in cMVD, we also found that Ad-null-EPC transfusion improved the relative CBF of peri-infarct area (Day 2, *P*<0.05; Day 7, *P*<0.01; Figure 8A) and transfusion of Ad-CXCR4 primed EPCs was more effective (Day 2, *P*<0.05; Day 7, *P*<0.01; Figure 8A). Meanwhile, the infarct volume was reduced (Day 2, *P*<0.05; Day 7, *P*<0.01; Figure 8B) after Ad-null-EPC infusion, and was able to be further decreased after the transfusion of Ad-CXCR4 primed EPCs at both day 2 and day 7 (*P*<0.01; Figure 8B). To evaluate the neurologic motor function, we measured neurologic deficit score at day 2 and 7 following MCAO and EPC infusion. We found that the neurologic deficit score was reduced in Ad-null-EPC group on day 7 (*P*<0.01; Figure 8C). Transfusion of Ad-CXCR4 primed EPCs improved neurologic motor function as early as on day 2 and had better efficacy on day 7 (*P*<0.01; Figure 8C).

### Infusion of Ad-CXCR4 Primed EPCs Enhances the Efficacy in Promoting Angiogenesis and Neurogenesis

**Figure 9:**
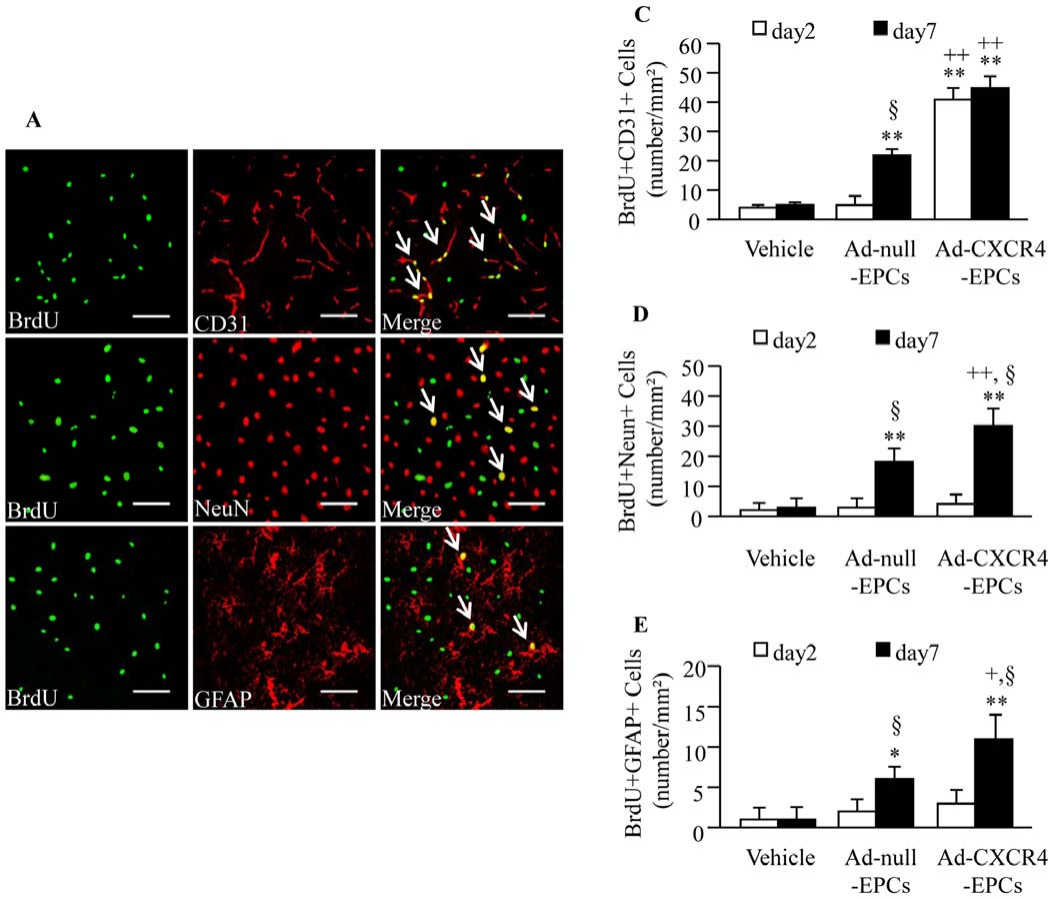
Infusion of Ad-CXCR4-EPCs increases angiogenesis and neurogenesis in db/db mice following ischemic stroke. (A) Representative pictures of angiogenesis (BrdU+CD31+), neurogenesis (BrdU+NeuN+) and glia cell genesis (BrdU+GFAP+) in the peri-infarct area 7 days after Ad-CXCR4-EPC treatment. Scale bar: 50 µm. Histogram showing the number of BrdU+CD31+ (B), BrdU+NeuN+ (C) and BrdU+GFAP+ (D) cells in the peri-infarct area on day 2 and 7 in different therapeutic groups. *P<0.05, **P<0.01 *vs.* vehicle; ^+^ *P*<0.05, ^++^ *P*<0.01 *vs.* Ad-null-EPCs; ^§^ *P*<0.05 *vs.* day 2, n = 6/group. NeuN: neuronal nuclei; GFAP: glial fibrillary acidic protein; Ad-null-EPCs: EPCs transfected with Ad-null; Ad-CXCR4-EPCs: EPCs transfected with Ad-CXCR4.

Figure 9A shows representative pictures of angiogenesis (BrdU+CD31+), glial (BrdU+GFAP+) and neuronal (BrdU+NeuN+) regenesis in the brain of db/db mice. Data showed that Ad-null-EPC transfusion increased angiogenesis and neurogenesis on day 7 (*P*<0.05 or 0.01; Figure 9B–D) without significant changes on day 2. Moreover, transfusion of Ad-CXCR4 primed EPCs promoted angiogenesis as early as day 2 (*P*<0.01), and had better efficacy in promoting angiogenesis and neurogenesis on day 7 (*P*<0.01 or 0.05; Figure 9B–D).

## Discussion

There are three major findings in this present study. Firstly, we found that the expression of SDF-1α/CXCR4 axis is dysregulated in the brain of db/db diabetic mice at basal and in response to ischemic stroke. Secondly, we illustrated that Ad-CXCR4 primed EPCs display resistance to HG-induced EPC dysfunction and apoptosis through activation of CXCR4 downstream PI3K/Akt/eNOS signal pathway. Thirdly, we demonstrated that infusion of Ad-CXCR4 primed EPCs acquires enhanced efficacy in reducing ischemic injury as well as promoting recovery.

The SDF-1α regulates tissue/organ homeostasis through the CXCR4 receptor expressed in hematopoietic progenitors. In the present study, we found that brain SDF-1α expression is reduced at basal and that ischemia-induced up-regulation of brain SDF-1α and CXCR4 are less in db/db mice. To our knowledge, this is the first evidence showing the dysregulation of SDF-1α/CXCR4 axis in the brain of an animal model with diabetes. A previous report showed that the expression of SDF-1α and CXCR4 is up-regulated in the tunica media of the thoracic aortas in streptozotocin-induced type-1 diabetes [24]. Although both type-1 and type-2 diabetic models have hyperglycemia, we tentatively attribute this inconsistence to different animal models and/or tissues used for the study. Nevertheless, our data are supported by other previous reports showing that the SDF-1α/CXCR4 axis is down-regulated in the wounds of db/db type-2 diabetic mice [35], [36]. The db/db model used in our study has hyperglycemia, obesity and dyslipidemia and insulin resistance [15]. Therefore, it deserves further investigation on if other factors besides hyperglycemia can also lead to the impairment of SDF-1α/CXCR4. Our *in vitro* data showed that HG down-regulated CXCR4 expression in EPCs, suggesting that hyperglycemia per se can impair the SDF-1α/CXCR4 axis [30], [37]. Furthermore, we found that HG impaired EPC migration and tube formation function, and induced EPC apoptosis. Although diabetes is a stage of severe inflammation and oxidative stress, and the *in vitro* study with high glucose cannot fully mimic the situation in *in vivo*, our results are supported by the reports from others [30], [23] showing that HG induced EPC senescence and dysfunction. Current data are also in agreement with our previous findings showing lower level of cEPCs and less cerebral microvascular density in db/db mice [3]. Therefore, hyperglycemia should be one of the mechanisms for EPC dysfunction in diabetes. On the other hand, evidence suggests that leptin can promote the mobilization of vascular progenitors from the bone marrow [38], [39]. The db/db mice possess an inactivating gene mutation in leptin receptor which could help to interpret our current findings in db/db mice.

In addition, we found that the levels of plasma SDF-1α and circulating CD34+CXCR4+ cells are reduced in db/db mice which are consistent with previous findings in diabetic patients [40]. Since the SDF-1α/CXCR4 interaction triggers several intracellular signals including MAPKs, PI3K and the serine/threonine kinase Akt, which modulate cell migration, proliferation and apoptosis [11], we investigated the implication of SDF-1α/CXCR4 axis in EPC function and survival in HG experiments. As expected, we found that Ad-CXCR4 transfection protects EPCs from HG-induced dysfunction and apoptosis. The underlying mechanism could be the activation of CXCR4 downstream PI3K/Akt/eNOS signal pathway since PI3K or eNOS inhibitor abolishes or partially blocks these protective effects. These results are in agreement with previous observations showing that SDF-1α/CXCR4 interaction mediates EPC migration via Akt and eNOS phosphorylation [41]. Collectively, our data suggest that the SDF-1α/CXCR4 axis is impaired at multiple sites (brain and EPCs) in diabetes, which might have implications in cerebral ischemic damage and repair (enlarged injury and delayed repair); targeting on the dysfunction of SDF-1α/CXCR4 axis could offer a new avenue for treating ischemic stroke in diabetes.

EPCs have been found to differentiate into ECs and contribute to angiogenic repair [42], [43]. A recent report demonstrated that transplantation of EPCs reduces infarct volume in ischemic stroke mice [7]. Yang et al [44] also demonstrated that CD34+ cells could represent a functional EPC population in bone marrow and have beneficial therapeutic effects in myocardial infarction. In patients with diabetes and db/db diabetic mice, circulating EPCs are reduced in number and are dysfunctional [3]–[5]. Our previous study demonstrates that transfusion of EPCs from non-diabetic sources has beneficial effect on ischemic stroke [3]. Previous reports by others also suggest that EPCs be useful for therapeutic purposes in diabetes [21], [45]. Because of the metabolic factors (such as hyperglycemia, hyperlipemia, etc) changes in diabetes, the efficacy of EPC-based therapy may be limited. This evidence provides a good rationale for using *in vitro* primed EPC in treating ischemic stroke in diabetes. On the other hand, this evidence suggests the control of metabolic factors in diabetes should be of importance to maximize the efficacy of EPC-based therapy. Here, we conducted *in vivo* studies to evaluate whether Ad-CXCR4 primed EPCs could enhance the benefits of EPC transfusion on treating ischemic stroke in the db/db diabetic mice. In agreement with previous reports showing transfusion of CXCR4 over-expressing EPCs or cardiac progenitor cells has better efficacy than CXCR4 low-expressing cells in treating hindlimb ischemia and myocardial infarction [14], [46], our data showed that Ad-CXCR4 primed EPCs have better effects over EPCs in alleviating cerebral damage (decreasing the infarct volume, improving neurologic deficits) and promoting cerebral repair (increasing cMVD, angiogenesis and neurogenesis). Angiogenesis is a vital component of tissue repair processes. EPCs are believed to play an important role in angiogenesis which represents an important endogenous tissue repair mechanism. The underlying mechanisms of EPCs promoting angiogenesis have been demonstrated. One is that EPCs physically participate in angiogenesis by incorporating and differentiating into matured ECs. The other is that EPCs secrete angiogenic factors promoting the proliferation and survival of resident ECs [6]. In this study, we measured newly generated ECs (CD31+BrdU+ cells) for the index of angiogenesis as commonly used by others [18], [47]. We found that the level of CD31+BrdU+ cells was increased in the peri-infarct area after EPC infusion, and more seen after infusion of CXCR4 primed EPCs. Our data demonstrate that CXCR4 over-expressing EPC further increases angiogenic repair following ischemic stroke.

The EPCs are thought to be a mixture of progenitor cells and mononuclear cells. At present, isolation of pure population of EPCs is challenging and the phenotypic characterization of the different types of EPC is currently an open issue with debate [48]. However, the generally accepted definition of EPCs is based on the expression of surface markers including CD34, CD133 and KDR [49]. In this study, we cultured EPCs for 7 days and characterized EPCs as CD34+VEGFR2+ cells. We found the percentage of CD34+VEGFR2+ cells was about 88.5%, suggesting most of them are EPCs. Moreover, the EPC isolation and culture techniques are also important to obtain the high purity EPCs. We isolated BM MNCs by gradient density separation method. BM MNCs were then plated on fibronectin-coated 24-well plates and grown in endothelial cell basal medium-2 containing EPC growth cytokine cocktails in favor of the persistence of EPCs at the expense of other cell lines [21], [50]. After 3 days of culture, non-adherent cells were removed by washing with PBS to avoid contamination of mononuclear cells. Therefore, we believe the observed effects are attributed to the transfused EPCs, rather than the CD45+ mononuclear cells.

Our *in vitro* EPC culture and *in vivo* animal studies are in a good agreement for supporting the beneficial effects of Ad-CXCR4 primed EPCs in treating diabetic stroke. Firstly, Ad-CXCR4 transfection protects EPCs from HG induced apoptosis resulting in increased level of circulating EPCs. Secondly, Ad-CXCR4 transfection prevents EPCs from HG-induced dysfunction (migration and tube formation) and leads to the promotion of angiogenesis. Transfusion of Ad-CXCR4 primed EPCs increases angiogenesis in peri-infarct area as early as day 2, whereas transfusion of EPCs shows this effect on day 7. However, we still observed an increase in cMVD and relative CBF on day 2 in EPC treatment group. Although the underline mechanism is unclear, we tentatively attribute it to the protective effect of EPCs, which secrete angiogenic factors promoting the proliferation and survival of resident ECs. Another major finding of our study is that Ad-CXCR4 primed EPCs are more effective than non primed EPCs in promoting cerebral repair processes. This is evidenced by increased angiogenesis, glia and neuron regenesis on day 7 in the Ad-CXCR4 primed EPC treatment group.

In summary, the present study demonstrates that transfusion of Ad-CXCR4 primed EPCs may be a novel approach for enhancing therapeutic benefits for ischemic stroke in diabetes. Over-expression of CXCR4 in EPCs prevents the deleterious effects of HG on EPC function and apoptosis via PI3K/Akt/eNOS signaling pathway which could be the underlying mechanism for the beneficial effects of Ad-CXCR4 primed EPC transfusion. Here, we want to point out that this study did not determine the level of EPC incorporation into endothelium, the level of local SDF-1α after EPC transfusion, and the paracrine effect of EPCs. These in-depth studies deserve future investigation.

## References

[1] Werner N, Nickenig G (2006) Influence of cardiovascular risk factors on endothelial progenitor cells: limitations for therapy? Arterioscler Thromb Vasc Biol 26: 257–266. 16322535 10.1161/01.ATV.0000198239.41189.5d

[2] Quirici N, Soligo D, Caneva L, Servida F, Bossolasco P, et al (2001) Differentiation and expansion of endothelial cells from human bone marrow CD133(+) cells. Br J Haematol 115: 186–194. 11722432 10.1046/j.1365-2141.2001.03077.x

[3] Chen J, Chen S, Chen Y, Zhang C, Wang J, et al (2011) Circulating endothelial progenitor cells and cellular membrane microparticles in db/db diabetic mouse: possible implications in cerebral ischemic damage. Am J Physiol Endocrinol Metab 301: E62–E71. 21505143 10.1152/ajpendo.00026.2011 PMC3129837

[4] Fadini GP, Miorin M, Facco M, Bonamico S, Baesso I, et al (2005) Circulating endothelial progenitor cells are reduced in peripheral vascular complications of type 2 diabetes mellitus. J Am Coll Cardiol 45: 1449–1457. 15862417 10.1016/j.jacc.2004.11.067

[5] Tepper OM, Galiano RD, Capla JM, Kalka C, Gagne PJ, et al (2002) Human endothelial progenitor cells from type II diabetics exhibit impaired proliferation, adhesion, and incorporation into vascular structures. Circulation 106: 2781–2786. 12451003 10.1161/01.cir.0000039526.42991.93

[6] Kalka C, Masuda H, Takahashi T, Kalka-Moll WM, Silver M, et al (2000) Transplantation of ex vivo expanded endothelial progenitor cells for therapeutic neovascularization. Proc Natl Acad Sci U S A 97: 3422–3427. 10725398 10.1073/pnas.070046397 PMC16255

[7] Fan Y, Shen F, Frenzel T, Zhu W, Ye J, et al (2010) Endothelial progenitor cell transplantation improves long-term stroke outcome in mice. Ann Neurol 67: 488–497. 20437584 10.1002/ana.21919 PMC3026588

[8] De FE, Porcelli D, Torella AR, Straino S, Iachininoto MG, et al (2004) SDF-1 involvement in endothelial phenotype and ischemia-induced recruitment of bone marrow progenitor cells. Blood 104: 3472–3482. 15284120 10.1182/blood-2003-12-4423

[9] Yamaguchi J, Kusano KF, Masuo O, Kawamoto A, Silver M, et al (2003) Stromal cell-derived factor-1 effects on ex vivo expanded endothelial progenitor cell recruitment for ischemic neovascularization. Circulation 107: 1322–1328. 12628955 10.1161/01.cir.0000055313.77510.22

[10] Tepper OM, Carr J, Allen RJ Jr, Chang CC, Lin CD, et al (2010) Decreased circulating progenitor cell number and failed mechanisms of stromal cell-derived factor-1alpha mediated bone marrow mobilization impair diabetic tissue repair. Diabetes 59: 1974–1983. 20484135 10.2337/db09-0185 PMC2911062

[11] Ganju RK, Brubaker SA, Meyer J, Dutt P, Yang Y, et al (1998) The alpha-chemokine, stromal cell-derived factor-1alpha, binds to the transmembrane G-protein-coupled CXCR-4 receptor and activates multiple signal transduction pathways. J Biol Chem 273: 23169–23175. 9722546 10.1074/jbc.273.36.23169

[12] Zemani F, Silvestre JS, Fauvel-Lafeve F, Bruel A, Vilar J, et al (2008) Ex vivo priming of endothelial progenitor cells with SDF-1 before transplantation could increase their proangiogenic potential. Arterioscler Thromb Vasc Biol 28: 644–650. 18239152 10.1161/ATVBAHA.107.160044

[13] Zhang D, Fan GC, Zhou X, Zhao T, Pasha Z, et al (2008) Over-expression of CXCR4 on mesenchymal stem cells augments myoangiogenesis in the infarcted myocardium. J Mol Cell Cardiol 44: 281–292. 18201717 10.1016/j.yjmcc.2007.11.010 PMC2601571

[14] Oh BJ, Kim DK, Kim BJ, Yoon KS, Park SG, et al (2010) Differences in donor CXCR4 expression levels are correlated with functional capacity and therapeutic outcome of angiogenic treatment with endothelial colony forming cells. Biochem Biophys Res Commun 398: 627–633. 20599766 10.1016/j.bbrc.2010.06.108

[15] Mantzoros CS, Moschos SJ (1998) Leptin: in search of role(s) in human physiology and pathophysiology. Clin Endocrinol (Oxf) 49: 551–567. 10197068 10.1046/j.1365-2265.1998.00571.x

[16] Chen S, Li G, Zhang W, Wang J, Sigmund CD, et al (2009) Ischemia-induced brain damage is enhanced in human renin and angiotensinogen double-transgenic mice. Am J Physiol Regul Integr Comp Physiol 297: R1526–R1531. 19759335 10.1152/ajpregu.91040.2008 PMC2777773

[17] Foubert P, Matrone G, Souttou B, Lere-Dean C, Barateau V, et al (2008) Coadministration of endothelial and smooth muscle progenitor cells enhances the efficiency of proangiogenic cell-based therapy. Circ Res 103: 751–760. 18723447 10.1161/CIRCRESAHA.108.175083

[18] Shyu WC, Lin SZ, Yang HI, Tzeng YS, Pang CY, et al (2004) Functional recovery of stroke rats induced by granulocyte colony-stimulating factor-stimulated stem cells. Circulation 110: 1847–1854. 15381647 10.1161/01.CIR.0000142616.07367.66

[19] Yokoi H, Yamada H, Tsubakimoto Y, Takata H, Kawahito H, et al (2010) Bone marrow AT1 augments neointima formation by promoting mobilization of smooth muscle progenitors via platelet-derived SDF-1{alpha}. Arterioscler Thromb Vasc Biol 30: 60–67. 19834109 10.1161/ATVBAHA.109.192161

[20] Cui X, Chen J, Zacharek A, Roberts C, Yang Y, et al (2009) Nitric oxide donor up-regulation of SDF1/CXCR4 and Ang1/Tie2 promotes neuroblast cell migration after stroke. J Neurosci Res 87: 86–95. 18711749 10.1002/jnr.21836 PMC2606920

[21] Marrotte EJ, Chen DD, Hakim JS, Chen AF (2010) Manganese superoxide dismutase expression in endothelial progenitor cells accelerates wound healing in diabetic mice. J Clin Invest 120: 4207–4219. 21060152 10.1172/JCI36858 PMC2993576

[22] Ingram DA, Lien IZ, Mead LE, Estes M, Prater DN, et al (2008) In vitro hyperglycemia or a diabetic intrauterine environment reduces neonatal endothelial colony-forming cell numbers and function. Diabetes 57: 724–731. 18086900 10.2337/db07-1507

[23] Chen YH, Lin SJ, Lin FY, Wu TC, Tsao CR, et al (2007) High glucose impairs early and late endothelial progenitor cells by modifying nitric oxide-related but not oxidative stress-mediated mechanisms. Diabetes 56: 1559–1568. 17389326 10.2337/db06-1103

[24] Jie W, Wang X, Zhang Y, Guo J, Kuang D, et al (2010) SDF-1alpha/CXCR4 axis is involved in glucose-potentiated proliferation and chemotaxis in rat vascular smooth muscle cells. Int J Exp Pathol 91: 436–444. 20586815 10.1111/j.1365-2613.2010.00720.x PMC3003841

[25] Mendoza-Naranjo A, Cormie P, Serrano AE, Wang CM, Thrasivoulou C, et al. (2012) Overexpression of the gap junction protein Cx43 as found in diabetic foot ulcers can retard fibroblast migration. Cell Biol Int. 10.1042/CBI20110628 22455314

[26] Kitahata Y, Nunomura S, Terui T, Ra C (2010) Prolonged culture of mast cells with high-glucose medium enhances the Fc epsilon RI-mediated degranulation response and leukotriene C4 production. Int Arch Allergy Immunol 152 Suppl 1 22–31. 20523060 10.1159/000312122

[27] Ghorbani A, Omrani GR, Hadjzadeh MA, Varedi M (2011) Effects of rat C-peptide-II on lipolysis and glucose consumption in cultured rat adipose tissue. Exp Clin Endocrinol Diabetes 119: 343–347. 21553365 10.1055/s-0031-1275662

[28] Zheng H, Dai T, Zhou B, Zhu J, Huang H, et al (2008) SDF-1alpha/CXCR4 decreases endothelial progenitor cells apoptosis under serum deprivation by PI3K/Akt/eNOS pathway. Atherosclerosis 201: 36–42. 18384792 10.1016/j.atherosclerosis.2008.02.011

[29] Chen TG, Chen JZ, Wang XX (2006) Effects of rapamycin on number activity and eNOS of endothelial progenitor cells from peripheral blood. Cell Prolif 39: 117–125. 16542347 10.1111/j.1365-2184.2006.00375.x PMC6495845

[30] Hamed S, Brenner B, Abassi Z, Aharon A, Daoud D, et al (2010) Hyperglycemia and oxidized-LDL exert a deleterious effect on endothelial progenitor cell migration in type 2 diabetes mellitus. Thromb Res 126: 166–174. 20347119 10.1016/j.thromres.2010.03.002

[31] Leu S, Sun CK, Sheu JJ, Chang LT, Yuen CM, et al (2011) Autologous bone marrow cell implantation attenuates left ventricular remodeling and improves heart function in porcine myocardial infarction: An echocardiographic, six-month angiographic, and molecular-cellular study. Int J Cardiol 150: 156–168. 20466442 10.1016/j.ijcard.2010.03.007

[32] Yang G, Chan PH, Chen J, Carlson E, Chen SF, et al (1994) Human copper-zinc superoxide dismutase transgenic mice are highly resistant to reperfusion injury after focal cerebral ischemia. Stroke 25: 165–170. 8266365 10.1161/01.str.25.1.165

[33] Girouard H, Lessard A, Capone C, Milner TA, Iadecola C (2008) The neurovascular dysfunction induced by angiotensin II in the mouse neocortex is sexually dimorphic. Am J Physiol Heart Circ Physiol 294: H156–H163. 17982007 10.1152/ajpheart.01137.2007

[34] Borlongan CV, Lind JG, llon-Carter O, Yu G, Hadman M, et al (2004) Bone marrow grafts restore cerebral blood flow and blood brain barrier in stroke rats. Brain Res 1010: 108–116. 15126123 10.1016/j.brainres.2004.02.072

[35] Bermudez DM, Xu J, Herdrich BJ, Radu A, Mitchell ME, et al (2011) Inhibition of stromal cell-derived factor-1alpha further impairs diabetic wound healing. J Vasc Surg 53: 774–784. 21211927 10.1016/j.jvs.2010.10.056 PMC3058337

[36] Badillo AT, Chung S, Zhang L, Zoltick P, Liechty KW (2007) Lentiviral gene transfer of SDF-1alpha to wounds improves diabetic wound healing. J Surg Res 143: 35–42. 17950070 10.1016/j.jss.2007.03.051

[37] Ceradini DJ, Yao D, Grogan RH, Callaghan MJ, Edelstein D, et al (2008) Decreasing intracellular superoxide corrects defective ischemia-induced new vessel formation in diabetic mice. J Biol Chem 283: 10930–10938. 18227068 10.1074/jbc.M707451200 PMC2447622

[38] Schroeter MR, Stein S, Heida NM, Leifheit-Nestler M, Cheng IF, et al (2012) Leptin promotes the mobilization of vascular progenitor cells and neovascularization by NOX2-mediated activation of MMP9. Cardiovasc Res 93: 170–180. 22065732 10.1093/cvr/cvr275

[39] Schroeter MR, Leifheit M, Sudholt P, Heida NM, Dellas C, et al (2008) Leptin enhances the recruitment of endothelial progenitor cells into neointimal lesions after vascular injury by promoting integrin-mediated adhesion. Circ Res 103: 536–544. 18658052 10.1161/CIRCRESAHA.107.169375

[40] Egan CG, Lavery R, Caporali F, Fondelli C, Laghi-Pasini F, et al (2008) Generalised reduction of putative endothelial progenitors and CXCR4-positive peripheral blood cells in type 2 diabetes. Diabetologia 51: 1296–1305. 18286257 10.1007/s00125-008-0939-6

[41] Zheng H, Fu G, Dai T, Huang H (2007) Migration of endothelial progenitor cells mediated by stromal cell-derived factor-1alpha/CXCR4 via PI3K/Akt/eNOS signal transduction pathway. J Cardiovasc Pharmacol 50: 274–280. 17878755 10.1097/FJC.0b013e318093ec8f

[42] Asahara T, Murohara T, Sullivan A, Silver M, van der ZR, et al (1997) Isolation of putative progenitor endothelial cells for angiogenesis. Science 275: 964–967. 9020076 10.1126/science.275.5302.964

[43] Asahara T, Masuda H, Takahashi T, Kalka C, Pastore C, et al (1999) Bone marrow origin of endothelial progenitor cells responsible for postnatal vasculogenesis in physiological and pathological neovascularization. Circ Res 85: 221–228. 10436164 10.1161/01.res.85.3.221

[44] Yang J, Ii M, Kamei N, Alev C, Kwon SM, et al. (2011) CD34+ cells represent highly functional endothelial progenitor cells in murine bone marrow. PLoS One 6: e20219. PONE-D-10–06134 [pii]. doi:10.1371/journal.pone.0020219. 10.1371/journal.pone.0020219 PMC3105013 21655289

[45] Cheng Y, Guo S, Liu G, Feng Y, Yan B, et al. (2012) Transplantation of bone marrow-derived endothelial progenitor cells attenuates myocardial interstitial fibrosis and cardiac dysfunction in streptozotocin-induced diabetic rats. Int J Mol Med. doi:10.3892/ijmm.2012.1083. 10.3892/ijmm.2012.1083 22859217

[46] Tang YL, Zhu W, Cheng M, Chen L, Zhang J, et al (2009) Hypoxic preconditioning enhances the benefit of cardiac progenitor cell therapy for treatment of myocardial infarction by inducing CXCR4 expression. Circ Res 104: 1209–1216. 19407239 10.1161/CIRCRESAHA.109.197723 PMC2756190

[47] Kawada H, Takizawa S, Takanashi T, Morita Y, Fujita J, et al. (2006) Administration of hematopoietic cytokines in the subacute phase after cerebral infarction is effective for functional recovery facilitating proliferation of intrinsic neural stem/progenitor cells and transition of bone marrow-derived neuronal cells. Circulation 113: 701–710. 113/5/701 [pii]; doi:10.1161/CIRCULATIONAHA.105.563668. 10.1161/CIRCULATIONAHA.105.563668 16461843

[48] Prater DN, Case J, Ingram DA, Yoder MC (2007) Working hypothesis to redefine endothelial progenitor cells. Leukemia 21: 1141–1149. 2404676 [pii]; doi:10.1038/sj.leu.2404676 10.1038/sj.leu.2404676 17392816

[49] Peichev M, Naiyer AJ, Pereira D, Zhu Z, Lane WJ, et al (2000) Expression of VEGFR-2 and AC133 by circulating human CD34(+) cells identifies a population of functional endothelial precursors. Blood 95: 952–958. 10648408

[50] Sen S, McDonald SP, Coates PT, Bonder CS (2011) Endothelial progenitor cells: novel biomarker and promising cell therapy for cardiovascular disease. Clin Sci (Lond) 120: 263–283. CS20100429 [pii]; doi:10.1042/CS20100429. 10.1042/CS20100429 21143202

